# The Perverts

**Published:** 1902-10

**Authors:** 


THE PERVERTS. By WILLIAM LEE HOWARD, M. D. Duodecimo, pp. 388. New York: G. W. Dillingham Co. 1902. [Price, $1.50.]

Here is a queer book; a book with an insight into life—the life of the underworld; and, although Dr. Howard has endeavored to clothe a scientific subject in a narrative gown, trimmed with anecdote and jeweled with the events of life in the slums, he has made neither a valuable scientific work, nor a brilliant novel. The elements of each are all there. A professional novelist would take the material in The Perverts and make of it one of the most amazing stories of modern times and all he would have to do would be to edit the present work as it stands. Dr. Howard has seen life in the slums as it really exists and he faithfully portrays it; yet it must be acknowledged that in some parts of the book there is an apparent weakness of construction—one might almost be tempted to say amateurish.

But the story, as a whole, is strong; its plot is well sustained, and there are times when one could easily imagine he were reading the pen picture of some one of the really great masters of modern realism. The descriptive character work is exquisitely real and in places there are startling pictures of life in the slums of New York with a cheap, room-rent, prostitute as the central character—a woman whose better self had not been entirely strangled by the life she led; a woman who, in spite of snuffchewing, cheap and spasmodic drunks and a cog-wheel life has not lost her respect for decency; in fact, just such a woman as one who has seen anything of slum life will recognise. For this reason, if for no other, the book is heartily recommended to the professional reformers of the better world; to the bands of well-meaning and respectable women workers in the cause of a higher christianity who do rescue work, serve on committees for the reclamation of lost and loosened souls, and wonder at the utter depravity of lost womanhood which sneers at tracts, rails at beef-stew suppers with ecclesiastic trimmings, turns in illy-disguised disgust from protestations of friendship and sisterhood which are offered from across the street, and repels half-hearted advances because she knows they are not sincere. It will show them that there is good in a fallen woman; it will show them that a woman, whose sin is in many cases the sin of necessity, wears a jewel which is rare even in highly respected, if not wholly respectable circles, where the sin of the outcast is the pastime of the saint—a jewel which a doctor will invariably find in the slums—gratitude. Those who expect to find in The Perverts a series of stories of warped sexual instinct, something after the fashion of Krafft-Ebbing's much sought after work of art, will be disappointed. There isn't a thing nasty in it from cover to cover. It is a clean book. The story concerns a family of perverts, one of whom, a doctor, is an alcoholic. His sisters are other types and they are all splendidly pictured and admirably described. Told in narrative form the story is interesting—especially so to the scientific mind. It is intensely dramatic in places and the descriptive work is of such a character that Dr. Howard should write a novel without any science in it. He has the true novelist's faculty for painting pen pictures of a most charming type. By all means read The Perverts. You will be the better for it—it will teach you something.

